# Supplementary material for: Voluntary medical male circumcision in selected provinces in South Africa: Outcomes from a programmatic setting
Source: PLoS One. 2022 Sep 23;17(9):e0270545. doi: 10.1371/journal.pone.0270545 (PMC9506619; doi:10.1371/journal.pone.0270545)
Supplement: Attachment — Submitted filename: PLOS One VMMC Paper.docx [file pone.0270545.s001.docx]

**Reviewer’s comments**

Manuscript Title: Voluntary Medical Male Circumcision (VMMC) in Selected Provinces in South Africa: Outcomes from a Programmatic Setting

Manuscript ID Number: PONE-D-21-05600

**Overall comment**

This is a very relevant manuscript with a large dataset, highlighting experiences from a routine program setting. The discussion section is relatively weak and needs to be strengthened before the manuscript is published

**General Comments on the sections**

Title – relevant to the topic presented

Abstract – well-presented summarizing the key findings outlined in the manuscript

Methods – study design is relevant to the study settings. Clear description of participants, procedures and data analysis methods

Results – the section is well narrated and the tables and illustrations elaborate the findings well.

Discussion – current discussion section is very general. It is lacking explicit recommendations or key programmatic implications from the findings of the manuscript. There is a wide array of relevant issues that could be included in this section and are currently missing. The authors just describe the overall findings but do not really discuss the implications – how do these findings help donors, funders, national programs or other implementers – to improve VMMC programming? Critical issues in VMMC global community include age prioritization, targeted HIV testing, sustainability of services with the decreasing donor funds, innovative demand creation approaches, underreporting of AEs, important SAEs such as glans injuries or urethral fistulae which are being tracked by WHO and PEPFAR, VMMC devices etc. It is a missed opportunity not to make recommendations on at least a few of these issues, given the fact that this was a large-scale review of programmatic data.

**Specific comments to be addressed**

Line 29 – role undetermined – check literature – there is very clear evidence role of VMMC in HIV prevention even beyond the RCTs - see <https://pubmed.ncbi.nlm.nih.gov/33043978/>

Line 84 – check sentence for correctness: was VMMC deferred if CD4 count was **above** or **below** 350?

Line 93 – It would be ideal to add a reference for MOVE such as:-(<https://www.malecircumcision.org/resource/considerations-implementing-models-optimizing-volume-and-efficiency-male-circumcision>)

Line 107 – why were clients under 10 years included in the database in the first place? Was this a data entry error or was it a violation of the PEPFAR policy for VMMC for HIV prevention which disallows circumcision for clients under 10years?

Line 118-123 – it will be more compelling to include number of clients circumcised by each methods of circumcision – i.e. Forceps Guided, Dorsal Slit, sleeve or device methods. If data is available, adverse events should be compared by method of circumcision to be able to compare with WHO recommendations and other studies done elsewhere

Line 120 – explain what happened to those who consented but did not undergo VMMC. Were they offered services later?

Line 132 – show the overall AE rate from 2012-2017 in the results paragraph. It is indicated later in Table 3 but it is important to show it early as it is a critical outcome indicator for the paper. Why were the rates calculated out of all VMMCs rather than out of those who returned for follow-up? Please provide an explanation

Table 1 – explain the meaning of “null” in the HIV status in Table 1

Line 131-138: Consider adding further details on types of AEs in this section, rather than just the three broad categories (infection-related, bleeding-related or insufficient skin removal). Current focus by both WHO and PEPFAR is on notifiable adverse events (NAEs) such as glans injuries and urethral fistulae which have programmatic implications. With such a large database, it is a huge opportunity to describe these critical SAEs (if data is available), rather than just the broad categories and will strengthen this manuscript.
