# Supplementary material for: Voluntary medical male circumcision in selected provinces in South Africa: Outcomes from a programmatic setting
Source: PLoS One. 2022 Sep 23;17(9):e0270545. doi: 10.1371/journal.pone.0270545 (PMC9506619; doi:10.1371/journal.pone.0270545)
Supplement: Attachment — Submitted filename: VMMC Paper- Responses to Reviewers.docx [file pone.0270545.s002.docx]

**Responses to the reviewer**

| **Specific comment to be addressed** | **Responses** |
| --- | --- |
| Line 29 – role undetermined – check literature – there is very clear evidence role of VMMC in HIV prevention even beyond the RCTs | This has been corrected in the manuscript line 30-32 |
| Line 84 – check sentence for correctness: was VMMC deferred if CD4 count was **above** or **below** 350? | This has been corrected in the manuscript line 89 |
| Line 93 – It would be ideal to add a reference for MOVE such as:-(<https://www.malecircumcision.org/resource/considerations-implementing-models-optimizing-volume-and-efficiency-male-circumcision)-> | Refence on MOVE teams has been added in the manuscript line 98 and reference No. 9 |
| Line 107 – why were clients under 10 years included in the database in the first place? Was this a data entry error or was it a violation of the PEPFAR policy for VMMC for HIV prevention which disallows circumcision for clients under 10years? | This has been addressed in line 112-113 in the manuscript.  Note: No clients below 10 years were circumcised in this program as they were no eligible. |
| Line 118-123 – it will be more compelling to include number of clients circumcised by each method of circumcision – i.e. Forceps Guided, Dorsal Slit, sleeve or device methods. If data is available, adverse events should be compared by method of circumcision to be able to compare with WHO recommendations and other studies done elsewhere. | This has been addressed in line 227-234 in the manuscript, under limitations |
| Line 120 – explain what happened to those who consented but did not undergo VMMC. Were they offered services later? | This is addressed in the manuscript in lines 139-144. |
| Line 132 – show the overall AE rate from 2012-2017 in the results paragraph. It is indicated later in Table 3 but it is important to show it early as it is a critical outcome indicator for the paper. Why were the rates calculated out of all VMMCs rather than out of those who returned for follow-up? Please provide an explanation. | This has been addressed in table 3 (Line 167) in the manuscript |
| Table 1 – explain the meaning of “null” in the HIV status in Table 1 | The “Null” category comprised clients with inconclusive results. This has been updated in the manuscript in table 1 and table 2 (Lines 137 and 151). |
| Line 131-138: Consider adding further details on types of AEs in this section, rather than just the three broad categories (infection-related, bleeding-related or insufficient skin removal). Current focus by both WHO and PEPFAR is on notifiable adverse events (NAEs) such as glans injuries and urethral fistulae which have programmatic implications. With such a large database, it is a huge opportunity to describe these critical SAEs (if data is available), rather than just the broad categories and will strengthen this manuscript. | This has been addressed in line 224-231 in the manuscript, under limitations |
