# Supplementary material for: Voluntary medical male circumcision in selected provinces in South Africa: Outcomes from a programmatic setting
Source: PLoS One. 2022 Sep 23;17(9):e0270545. doi: 10.1371/journal.pone.0270545 (PMC9506619; doi:10.1371/journal.pone.0270545)
Supplement: Attachment — Submitted filename: VMMC Observational cohort_manuscript__Revised_clean wm.docx [file pone.0270545.s003.docx]

**Voluntary Medical Male Circumcision in Selected Provinces in South Africa: Outcomes from a Programmatic Setting**

**Khumbulani Moyo^1^_,_ Nelson Igaba^1^_,_ Constance Wose Kinge^1^, Charles Chasela^1,2*^_,_ Motshana Phohole^1^, Skye Grove^1^, Caroline Makura^1^, Latisani Mudau^1^, Dirk Taljaard^3^, Dino Rech^3^, Arthi Ramkissoon^4±^, Catherine Searle^4^, Pappie Majuba^1^ and Ian Sanne^1,5^**

^1^Right to Care, Johannesburg, South Africa

^2^Department of Epidemiology and Biostatistics, School of Public Health, Faculty of Health Sciences, University of the Witwatersrand, Johannesburg, South Africa

**^3^**Centre for HIV and AIDS Prevention Studies (CHAPS), Johannesburg, South Africa

^4^Maternal Adolescent and Child Health (MatCH), Durban, South Africa

^5^Clinical HIV Research Unit, Department of Clinical Medicine, Faculty of Health Sciences, University of the Witwatersrand, Johannesburg, South Africa

***Correspondence:** charles.chasela@wits.ac.za Tel: +27-76-5240898

**±** Deceased

**Abstract**

**Introduction:** Voluntary medical male circumcision (VMMC) remains an effective biomedical intervention for HIV prevention in high HIV prevalence countries. In South Africa, United States Agency for International Development VMMC partners provide technical assistance to the Department of Health, at national and provincial levels in support of the establishment of VMMC sites as well as in providing direct VMMC services at site level since April 2012. We describe the outcomes of the Right to Care (RTC) VMMC program implemented in South Africa from 2012 to 2017.

**Methods:** This retrospective study was undertaken at RTC supported facilities across six provinces. Young males aged ≥10 years who presented at these facilities from 1 July 2012 to 31 September 2017 were included. Outcomes were VMMC uptake, HIV testing uptake and rate of adverse events. Using a de-identified observational database of these clients, summary statistics of the demographic characteristics and outcomes were calculated.

**Results:** There were a total 1,001,226 attendees of which 998, 213 (99.7%) were offered VMMC and had a median age of 15 years (IQR= 12–23 years). Of those offered VMMC, 99.6% (994,293) consented, 96.7% (965,370) were circumcised and the majority (46.3%) were from Gauteng province. HIV testing uptake was 71% with a refusal rate of 15%. Of the newly diagnosed HIV positives, 64% (6,371 / 9,972) referrals were made. The rate of adverse events (AEs, defined as bleeding, infection and insufficient skin removal) declined from 3.26% in 2012 to 1.17% in 2017. There was a reduction in infection-related AEs from 2448 (94.08%) in 2012 to 129 (46.9%) in 2017 of the total AEs reported each year over the five-year period.

**Conclusion:** There was a high VMMC uptake with a decline in AEs over time. Adolescent men contributed the most to the circumcised population, an indication that the young population access medical circumcision. HIV prevalence in the total population increased with increasing age, notably in clients above 25 years. Targeting young men below the age of 25 years will help to prevent HIV spread and accelerate reaching epidemic control.

**Key words: Male circumcision, Prevention, Adverse events, HIV testing**

**Introduction**

Voluntary medical male circumcision (VMMC) remains one of the interventions for HIV prevention in countries with high HIV prevalence. Medical male circumcision (MMC) is associated with a 60% reduction in the risk of female-to-male HIV transmission (1-3). Furthermore, VMMC programs are considered highly effective in reducing both HIV incidence and the cost of HIV prevention especially when targeting males between 20 to 25 years (4). Male circumcision only offers partial protection against HIV transmission, hence, MMC must be integrated with a comprehensive HIV prevention strategy, which includes treatment for sexually transmitted infections, HIV testing and counselling and promotion of safe sex practices (5, 6) and provision of pre-exposure prophylaxis (PrEP).

Traditional circumcision, also viewed as a “rite of passage into manhood” is a common practise among the Xhosa, Sotho, Pedi, Venda and Ndebele cultural groups of South Africa (1, 7). This “traditional rite” is usually performed in the months of June, July, November and December, commonly referred to as the circumcision season. The procedure is performed by traditional leaders without adequate infection control measures in place. As a result, traditional circumcision is often associated with multiple surgical complications and increased risk of injury and even death among young men and boys (7).

In 2012, USAID awarded Right to Care (RTC) a contract to implement a VMMC program across the provinces of Gauteng, KwaZulu-Natal (KZN), Free State, Limpopo, Mpumalanga and North West in South Africa. RTC implemented the VMMC program in partnership with the Centre for HIV/AIDS Prevention Studies (CHAPS), ANOVA Health Institute and Maternal Adolescent and Child Health (MatCH). In this paper, we report the outcomes of the VMMC program implemented across the six provinces from 2012 to 2017.

**Materials and Methods**

*Study design*

This was retrospective data analysis using routinely collected program data by the South African Department of Health USAID funded VMMC program for the period of 1 July 2012 to 30 September 2017.

*Study setting*

The VMMC program operated in 149 sites across selected districts in Gauteng, Free State, KwaZulu-Natal, Limpopo, Mpumalanga and North West Provinces. The districts include City of Johannesburg, City of Tshwane, Ekurhuleni, Sedibeng and West Rand for Gauteng; Fezile Dabi and Lejweleputswa for Free State; eThekwini, Ugu, uMgungundlovu, uMkhanyakude and Zululand for KwaZulu-Natal; Capricorn, Mopani and Vhembe for Limpopo; Ehlanzeni and Gert Sibande for Mpumalanga; and Bojanala Platinum for North West. The sites in Limpopo, Mpumalanga, Free State and North West Provinces cater mainly for the rural population while those in the Gauteng and Kwa-Zulu Natal Provinces cater for the urban population. Selection of sites was determined by the funder (USAID) and the South Africa Department of Health at national and provincial levels.

*Participants*

All males aged ≥10 years were eligible for circumcision. This age group is in line with the South African MMC National Guidelines (8). Written informed consent for MMC was required and given independently by all males aged ≥18 years. Boys aged 16-17 years provided assent to the circumcision procedure after being given information with the parent or legal guardian giving written informed consent. All boys aged 11-15 years required parental/guardian written informed consent to undergo MMC. The client gave assent, and the parent/guardian was required to be present on the day of the circumcision.

*Recruitment of participants*

Demand creation activities were undertaken through campaigns at community level, messaging through social media, radio, and other forms of media. All those willing were booked and referred to a facility for further screening, consenting and circumcision. At the facility, clients were recruited through walk- ins and referrals from other service points.

*Circumcision procedures*

Once a client was recruited at the facility, the client was registered and provided with general group education. Following group education, individual counselling and HIV testing was offered, and subsequently MMC to all eligible clients (**Figure 1)**.

**Figure 1: Patient Flow for VMMC at site of operation**

Pre-operative history taking, physical examination and circumcision procedures were carried out among clients who accepted and consented. VMMC was deferred for new HIV positive clients to when available, CD_4_ counts was < 350 cells/µL but referred for antiretroviral therapy (ART) initiation. For known HIV positive clients, an assessment of the client’s adherence to ART and retention in HIV care was conducted. The client was referred for care if he was lost to follow up. Eligibility for circumcision for such clients was based on recent CD_4_ ≥ 350 µ/L and/or viral load (VL) of Lower Than Detectable Limits (LTDL) with CD_4_ and VL results not more than six months old. Circumcision was deferred if VL > 1000 copies/ml regardless of CD_4_ value.

*VMMC site team composition*

The team composition was built to ensure optimization of volumes and efficiency in the delivery of services, a model called MOVE “Model for Optimizing Volume and Efficiency” (9). The MOVE team comprised of a Surgeon (Clinical Associate or Medical Officer), Professional Nurse (PN), two enrolled nurses, two counsellors, administration clerk, a cleaner and a driver.

*Post-operative follow ups*

Following the MMC procedure, follow up visits were scheduled to assess for any signs of AEs. The first assessment occurred immediately after the procedure to check for signs of bleeding or any event and if there was no problem, the client was discharged from the facility. The subsequent follow-up visits were scheduled on 2, 7- and 42-days post-circumcision.

*Data management and analysis*

Client data captured by RTC data capturers into RightMax (a cloud-based database for financial and programmatic reporting and monitoring purposes) for the period of 1 July 2012 to 30 September 2017 was retrieved and exported into Microsoft Excel. Client name and surname, date of birth, and ID or passport number as well as phone numbers were deleted. The de-identified data was then imported into Stata version 15 for further management and analysis. Non-eligible clients were excluded from the analysis where necessary. Proportions were calculated to describe the population characteristics as well as the outcomes and consenting, age categories, circumcised, HIV testing and status of all attendees including females.

*Ethical considerations*

Informed consent or assent was obtained from either the patients or the parents/guardians of the adolescents as part of the program requirements. Ethics clearance was obtained from the University of the Witwatersrand, Johannesburg Human Research Ethics Committee (# M150823).

**Results**

*Participants*

Out of 1,001,266 attendees, 99.7% (998,213) participants were offered VMMC and out of these 99. 6% (994,293) accepted and consented for VMMC. Out of those that consented, 97.2 (965,370) were circumcised (**Figure 2**). The median age of the participants was 15 years (IQR = 12–23 years) and 65.7% were between ages 10 and 19 years (n=655,490). Most clients were from Gauteng (46.3%), followed by Mpumalanga Province (20%) and most circumcisions were done between 2013 and 2017 (**Table 1**).

**Figure 2: Flow of participants for the VMMC program**

Table 1. Description of participants characteristics

| Characteristics | N | % |
| --- | --- | --- |
| Age (median, IQR) | 15 | 12–23 |
| Age Categories (%) |  |  |
| 10-14 | 449,683 | 45.05 |
| 15-19 | 205,807 | 20.62 |
| 20-24 | 121,723 | 12.19 |
| 25-29 | 89,184 | 8.93 |
| 30-34 | 59,053 | 5.92 |
| 35-39 | 34,600 | 3.47 |
| 40 and Older | 38,163 | 3.82 |
| Province (%) |  |  |
| Free State | 41,238 | 4.13 |
| Gauteng | 462,128 | 46.30 |
| KwaZulu-Natal | 145,801 | 14.61 |
| Limpopo | 94,875 | 9.50 |
| Mpumalanga | 199,380 | 19.97 |
| North West | 54,791 | 5.49 |
| Year (%) |  |  |
| 2012 | 81,941 | 8.21 |
| 2013 | 139,931 | 14.02 |
| 2014 | 200,515 | 20.09 |
| 2015 | 206,163 | 20.65 |
| 2016 | 188,268 | 18.86 |
| 2017 | 181,395 | 18.17 |
| *HIV testing offered (%) |  |  |
| Tested | 711,882 | 71.11 |
| Not tested (Known status) | 139,225 | 13.91 |
| Declined tested | 149,981 | 14.98 |
| * HIV Status (%) |  |  |
| Negative | 692,880 | 69.21 |
| Known negative | 115,638 | 11.55 |
| Positive | 18,673 | 1.87 |
| Known positive | 23,596 | 2.36 |
| Unknown | 150,301 | 15 |
| * Newly Diagnosed HIV (%) |  |  |
| Not referred to treatment | 2,503 | 13.40 |
| Referred to treatment | 16,170 | 86.60 |

* The number of HIV testing offered included female attendees but not males less than 10 years.

Clients who consented but did not undergo circumcision were those that either tested HIV positive on the day of circumcision and had CD4 <350 cell/uL or had one of the contra-indications as listed in the South African MMC guidelines (8). Those that tested HIV positive were referred for ART initiation and those with either infection, uncontrolled chronic illnesses or penile anatomical abnormalities were referred for further management as per the South African National Department of Health (NDoH) referral guidelines (8).

*HIV testing and linkage to care*

A total of 1,001,088 were offered an HIV test and 71.1% were tested, 15% declined the test and the rest had a known status. These included men and women who presented at the facility. Out of those newly diagnosed, referrals were undertaken in 86.6% (**Table 1**). The HIV prevalence increased with age with the highest among those ≥30 years (**Table 2**).

Table 2: HIV Status by age groups

| Age | HIV Status, n (%) | | | | | |
| --- | --- | --- | --- | --- | --- | --- |
|  | **Negative** | **Positive** | **Known Negative** | **Known Positive** | **Unknown** | **Total** |
| 10-14 | 283, 680 (63.06) | 2, 391 (0.53) | 48, 990 (10.89) | 3, 428 (0.76) | 111, 338 (25) | 449, 827 (100) |
| 15-19 | 157, 737 (76.51) | 1, 241 (0.60) | 32, 144 (15.59) | 1, 414 (0.69) | 13, 631 (7) | 206, 167 (100) |
| 20-24 | 95, 913 (78.39) | 1, 841 (1.50) | 15, 752 (12.87) | 1, 1141 (0.93) | 7, 698 (6) | 122, 347 (100) |
| 25-29 | 67, 195 (74.88) | 3, 725 (4.15) | 8, 778 (9.84) | 3, 217 (3.58) | 6, 823 (8) | 89, 738 (100) |
| 30-34 | 41, 117 (69.20) | 3, 939 (6.63) | 4, 696 (7.95) | 4, 692 (7.90) | 4, 976 (8) | 59, 420 (100) |
| 35-39 | 22, 688 (65.10) | 2, 668 (7.66) | 2, 396 (6.92) | 4, 222 (12.11) | 2, 876 (8) | 34, 850 (100) |
| 40 and older | 24, 550 (63.37) | 2, 868 (7.40) | 2, 882 (7.55) | 5, 4812(14.15) | 2, 957 (8) | 38, 739 (100) |
| Total | **692, 880 (69.21)** | **18, 673 (1.87)** | **115, 638 (11.55)** | **23, 596 (2.36)** | **150, 301 (15)** | **998, 214 (100)** |

*Adverse events (AEs)*

There were 10, 608 reported AEs between 2012 and 2017 with the highest numbers reported among clients aged 15-19 years (n=3, 048; 28.73%) (**Figure 3**). Out of these, 58.81% (n=6, 239) were infection-related followed by 8.30% (n=880) due to bleeding, 0.92% (n=98) were due to insufficient skin removal and 31.97% (n=3, 391) of the AEs had missing AE type. The rate of AEs reduced over time with the highest incidence of 71.7%, (n=2, 602) in the year 2012­ and 12% (n=2, 004) in 2013 (**Table 3**). There was a reduction in infection related AEs from 94.08% (2, 448) in 2012 to 46.9% (129) in 2017 of the total AEs reported each year over the five-year period (**Table 4**). Among all identified AEs, 4% (n=423) were device related, 32.70% (n=3, 469) were surgical related. However, 63.3% (n=6, 716) had missing technique of circumcision (**Table 4**).

**Figure 3**. Number of AEs identified by type per age group

**Table 3**: Adverse events rates

| Year | Timing | | Severity | | | Total AEs | VMMCs Done | Follow-up | AE Rate (%) |
| --- | --- | --- | --- | --- | --- | --- | --- | --- | --- |
|  | **Intra-operative** | **Post-operative** | **Mild** | **Moderate** | **Severe** |  |  |  |  |
| 2012 | 11 | 2, 591 | 0 | 1, 968 | 634 | 2, 602 | 79, 820 | 3, 631 | 71.7% |
| 2013 | 5 | 1, 999 | 0 | 1, 831 | 173 | 2, 004 | 136, 163 | 16, 745 | 12.0% |
| 2014 | 35 | 1, 382 | 0 | 1, 385 | 32 | 1, 417 | 194, 359 | 55, 614 | 2.5% |
| 2015 | 15 | 471 | 0 | 448 | 38 | 486 | 198, 587 | 81, 201 | 0.6% |
| 2016 | 1, 630 | 400 | 1, 488 | 452 | 90 | 2, 030 | 179, 904 | 106, 661 | 1.9% |
| 2017 | 1, 930 | 139 | 1, 528 | 391 | 150 | 2, 069 | 176, 537 | 72, 411 | 2.9% |
| Total | **3, 626** | **6, 982** | **3, 016** | **6, 475** | **1, 117** | **10, 608** | **965, 370** | **336, 263** | **3.2%** |

**Table 4**. Types of Adverse events from 2012-2017

| **Year** | **AE Type, n (%)** | | | | |
| --- | --- | --- | --- | --- | --- |
|  | **Bleeding related** | **Infection related** | **Insufficient Skin Removal** | **Missing** | **Total** |
| **2012** | 134 (5.15) | 2, 448 (94.08) | 20 (0.77) | 0 (0.00) | 2, 602 (100) |
| **2013** | 244 (12.18) | 1, 741 (86.88) | 19 (0.95) | 0 (0.00) | 2, 004 (100) |
| **2014** | 162 (11.43) | 1, 242 (87.65) | 13 (0.92) | 0 (0.00) | 1, 417 (100) |
| **2015** | 86 (17.70) | 373 (76.75) | 27 (5.56) | 0 (0.00) | 486 (100) |
| **2016** | 111 (5.47) | 306 (15.07) | 16 (0.79) | 1, 597 (78.67) | 2, 030 (100) |
| **2017** | 143 (6.91) | 129 (6.23) | 3 (0.14) | 1, 794 (86.71) | 2, 069 (100) |
| **Total** | **880 (8.30)** | **6, 239 (58.81)** | **98 (0.92)** | **3, 391(31.97)** | **10, 608 (100)** |

**Discussion**

In this paper, we report the outcomes and trends for a USAID funded VMMC program over a five–year period (2012-17) across six provinces in South Africa. A majority of mobilized VMMC eligible attendees were between the ages 10 to 24 years and were offered MMC. This is indicative of a young population seeking VMMC. A review of VMMC program in nine eastern and southern African countries including South Africa also found that the majority of clients were young men; 60% were older adolescents (aged 15 to 19 years) (10). In addition, the program reached the young adolescent men, who are sexually active and at risk for HIV acquisition. However, to achieve immediate impact in reduction of HIV incidence in these districts, the VMMC program needs to target males 15-29 years old. This is in line with WHO and NDoH guidance on maximize impact on VMMC in HIV prevention. Out of the six provinces, most circumcisions were undertaken in Gauteng Province with increasing number of circumcisions over time. The Thembisa modelling 2.0 estimates that most provinces in South Africa have not reached the VMMC saturation mark of circumcising 80% of males 15-49 years old. Limpopo province is the only province with an estimate circumcision saturation of more than 80% and this could be attributed to their culture practice of traditional male circumcision of young males as early as 10 years of age (11). The South African VMMC program will need to implement innovative ways of scaling up VMMC services to provinces with high HIV prevalence and low circumcision saturation like KwaZulu Natal and Mpumalanga.

There was a high acceptance of HIV testing among those not tested, with 14% known status and the rest declining testing. HIV prevalence was about 2% among the newly diagnosed and 3% among participants who were known positives. HIV prevalence was high among those above 25 years and positivity increased with age with a positivity rate of about 8% among those newly diagnosed and between the ages 35–39 and 12-14% above 40 years of age HIV prevalence among participants known positive. The 3% was lower compared to general HIV prevalence in South Africa which is 12.2% (12). However, the rate of positivity was high among the newly tested in the ages above 35 years. The refusal rate for HIV testing was 14% which is in line with findings from other studies where program data from nine southern and east African countries indicated HIV testing refusal among 13.5% of participants. (10). The higher HIV positivity rate in older men aged >20 years is indicates the need to focus efforts for VMMC and other HIV prevention modalities amongst this age group to create an impact on reduction of HIV incidence in these communities. The VMMC program targets young healthy HIV negative males to confer to them the benefit of reduced transmission of HIV from an HIV positive female partner by 60%.

The common AE was infection, followed by bleeding. However, the occurrence of AEs dropped from 71.7% in 2012 to 3.2 % in 2017. This finding is in agreement with data from other countries which have shown reducing trends in AEs over time, as providers become more proficient in the procedure. The reduction in infection-related AEs from 94.08% in 2012 to 46.9% in 2017 of the total number of AEs reported each year over the five-year period, indicates improvement in infection prevention practices and quality of care. The relatively higher rates of AEs and low rates of follow-up in our study could be attributed to challenges in documentation at facility level. Close monitoring and documentation are recommended to help program quality improvement. Our study showed that males circumcised by device technique were more likely to develop an AE (OR=6.98) compared to those circumcised by surgical technique. This finding corroborates those of other studies (13) which implies the need of proper training and mentorship for staff, and appropriate clients’ education and follow-up whenever device technique is implemented for MMC.

Majority of the identified AEs in our study were reported in the 15 to 19 years old age group, contrary to findings in other studies (13) where 10 to 14 year olds contributed most of the AEs especially those related to infection. The findings of our study could be attributed to the change in policy by the NDoH in 2016 requiring all clients 10-14 years old to be accompanied to the health facility by their parent/guardian on the day of male circumcision procedure and participate in health education for wound care at home. The high number of infection related AEs across all age groups indicates need for increased emphasis on interpersonal communication measures at health facilities, clients’ health education on wound hygiene while at home, education on avoiding use of herbal medicine and home remedies on the wound and returning for physical review of the wound as per programmatic schedule.

**Limitations**

The following limitations are noted. Firstly, being routine program data some important variables to inform relationships were missed. For example, the technique used during male circumcision procedure was only recorded as device method or surgical method. The surgical method was not disaggregated further into forceps guided, dorsal slit or sleeve resection methods. Furthermore, only three types of AEs were included (bleeding, infection, and insufficient skin removal) with 31.97% (n=3,391) missing information on type. Client follow-up was done but this was likely not well-captured in the system. However, the large data set offered a real-world experience thus maximizing generalizability, which often is a challenge in small data sets. Secondly, being routine data, there were several processing challenges due to data capturing, which may lead to misclassification, and subsequent bias. However, manual reviews were conducted, data was verified with source documents thereby improving the quality of the data, giving more representative and generalizable results.

In conclusion, there was high acceptance of circumcision and low HIV prevalence among the young men. While, most accepted HIV testing, the number of refusals is still high and requires intensified demand creation efforts. VMMC remains one of the major HIV preventive mechanisms and more demand will help to reach HIV epidemic control.

**Acknowledgments**

Kgagamatso Chimelwane and Lynette Stone for assisting with RightMax downloads

**Author contributions**

KM conceptualized the idea, facilitated access to RightMax, reviewed the results and the manuscript.

CSC reviewed and re-wrote the manuscript.

KS statistical analysis consolidation and reviewed the data and results.

CWK carried out statistical analysis and reviewed the manuscript.

CM designed the analysis plan, reviewed the data and data analysis.

All authors reviewed and approved the manuscript.

**Conflict of Interest**

No conflict of interest declared

**References**

1.	Auvert B, Lissouba P, Taljaard D, Geffen N, Fiamma A, Heywood M. Key facts on male circumcision. S Afr Med J. 2009;99(3):150-1.

2.	Bailey RC, Moses S, Parker CB, Agot K, Maclean I, Krieger JN, et al. Male circumcision for HIV prevention in young men in Kisumu, Kenya: a randomised controlled trial. Lancet. 2007;369(9562):643-56.

3.	Gray RH, Kigozi G, Serwadda D, Makumbi F, Watya S, Nalugoda F, et al. Male circumcision for HIV prevention in men in Rakai, Uganda: a randomised trial. Lancet. 2007;369(9562):657-66.

4.	Haacker M, Fraser-Hurt N, Gorgens M. Effectiveness of and Financial Returns to Voluntary Medical Male Circumcision for HIV Prevention in South Africa: An Incremental Cost-Effectiveness Analysis. PLoS Med. 2016;13(5):e1002012.

5.	Lagarde E, Dirk T, Puren A, Reathe RT, Bertran A. Acceptability of male circumcision as a tool for preventing HIV infection in a highly infected community in South Africa. Aids. 2003;17(1):89-95.

6.	Coates TJ, Richter L, Caceres C. Behavioural strategies to reduce HIV transmission: how to make them work better. Lancet. 2008;372(9639):669-84.

7.	Weiss HA, Quigley MA, Hayes RJ. Male circumcision and risk of HIV infection in sub-Saharan Africa: a systematic review and meta-analysis. Aids. 2000;14(15):2361-70.

8.	Health NDo. Pretoria: National Department of Health; 2016.

9.	WHO. Considerations for implementing models for optimizing the volume and efficiency of male circumcision services. Geneva: World Health Organization; 2010 [cited 2021. Available from:https://www.malecircumcision.org/sites/default/files/document_library/Considerations%20models.pdf.

10.	Voluntary medical male circumcision - southern and eastern Africa, 2010-2012. MMWR Morb Mortal Wkly Rep. 2013;62(47):953-7.

11.	Health NDo. Male circumcision percentage in South Africa by province Pretoria: Health Systems Trust; 2003 [updated 13 Sep 2015; cited 2021 9th August, 2021]. Available from: <https://indicators.hst.org.za/HIV%20and%20AIDS/HIV%20and%20AIDS/sahr_276/IND>.

12.	Zuma K, Shisana O, Rehle TM, Simbayi LC, Jooste S, Zungu N, et al. New insights into HIV epidemic in South Africa: key findings from the National HIV Prevalence, Incidence and Behaviour Survey, 2012. Afr J AIDS Res. 2016;15(1):67-75.

13.	Feldacker C, Bochner AF, Murenje V, Makunike-Chikwinya B, Holec M, Xaba S, et al. Timing of adverse events among voluntary medical male circumcision clients: Implications from routine service delivery in Zimbabwe. PLoS One. 2018;13(9):e0203292.
