# Supplementary material for: Voluntary medical male circumcision in selected provinces in South Africa: Outcomes from a programmatic setting
Source: PLoS One. 2022 Sep 23;17(9):e0270545. doi: 10.1371/journal.pone.0270545 (PMC9506619; doi:10.1371/journal.pone.0270545)
Supplement: Attachment — Submitted filename: Response to Reviewers.docx [file pone.0270545.s005.docx]

Dear Editor

Thanks for the review and comments. We have worked on an updated version and below are the specific responses with the place where the change has been made.

Responses to the reviewer

| Specific comment to be addressed | Answer |
| --- | --- |
| Include citation | This has been addressed in line 233 |
| Which device resulted in the AEs? I am sure authors are aware that PrePex has now been taken off the market. Does this recommendation still apply? | This has been addressed in line 233-236 |
| It is now 4 years since these data were collected. To what extent are the findings and conclusions still applicable/relevant? If still relevant, the paper could say in a couple of places, how the data compare to what is currently obtaining within the VMMC program. | This has been addressed in line 189-192, 207-209, 359-365 |

We look forward to the paper being published in your prestigious journal.

Yours Sincerely

Charles Chasela
