# Supplementary material for: Voluntary medical male circumcision in selected provinces in South Africa: Outcomes from a programmatic setting
Source: PLoS One. 2022 Sep 23;17(9):e0270545. doi: 10.1371/journal.pone.0270545 (PMC9506619; doi:10.1371/journal.pone.0270545)
Supplement: Attachment — Submitted filename: Responses to reviewer.docx [file pone.0270545.s007.docx]

**Responses to the reviewer**

| **Specific comment to be addressed** | **Answer** |
| --- | --- |
| Consistency to using 2 decimal points | Corrected from line 165-172 including table 3 last column (AE rate %) |
| References 2-4 | Replaced in line 33 and line 301-307 |
| References 5-20 | Changed from line 308-357 |
| Reference 7 | Replaced in line 43, 231 and 314-317 |
| Reference 8 | Replaced in line 45 and 318-319 |
| Reference 9 | Replaced in line 49 and 320-322 |
| Reference 10 | Replaced in line 75, 187, 249 and 323-325 |
| Reference 11 | Replaced in line 106 and 326-329 |
| Reference 12 | Replaced in line 183, 219 and 330-334 |
| Reference 13 | Replaced in line 187, 190, 193, 199 and 335-338 |
| Reference 14 | Replaced in line 204 and 339-341 |
| Reference 15 | Replaced in line 216 and 342-344 |
| Reference 16 | Replaced in line 229, 240, 245, 256 and 345-347 |
| Reference 17 | Replaced in line 235 and 348-350 |
| Reference 18 | Replaced in line 243 and 351-352 |
| Reference 19 | Replaced in line 253 and 353-355 |
| Reference 20 | Replaced in line 256 and 356-357 |
| Text correction | Line 183 |
| Text correction | Line 219 |
